# Gene Variants Determine Placental Transfer of Perfluoroalkyl Substances (PFAS), Mercury (Hg) and Lead (Pb), and Birth Outcome: Findings From the UmMuKi Bratislava-Vienna Study

**DOI:** 10.3389/fgene.2021.664946

**Published:** 2021-06-16

**Authors:** Claudia Gundacker, Klaudia Graf-Rohrmeister, Martin Gencik, Markus Hengstschläger, Karol Holoman, Petra Rosa, Renate Kroismayr, Ivo Offenthaler, Veronika Plichta, Theresa Reischer, Isabella Teufl, Wolfgang Raffesberg, Sigrid Scharf, Birgit Köhler-Vallant, Zoja Delissen, Stefan Weiß, Maria Uhl

**Affiliations:** ^1^Institute of Medical Genetics, Medical University of Vienna, Vienna, Austria; ^2^Semmelweis Frauenklinik, Vienna, Austria; ^3^Klinik Floridsdorf, Vienna, Austria; ^4^Medgene, Bratislava, Slovakia; ^5^University Hospital Bratislava-Ružinov, Bratislava, Slovakia; ^6^Department of Biochemical Engineering, University of Applied Sciences Technikum Wien, Vienna, Austria; ^7^Environment Agency Austria, Vienna, Austria; ^8^Austrian Agency for Food and Health Safety, Vienna, Austria

**Keywords:** placental transfer, birth outcome, perfluoroalkyl substances (PFAS), Bisphenol a (BPA), Mercury (Hg), lead (Pb), genotype-phenotype

## Abstract

Prenatal exposure to perfluoroalkyl substances (PFAS), bisphenol A (BPA), lead (Pb), total mercury (THg), and methylmercury (MeHg) can affect fetal development. Factors influencing placental transfer rate of these toxins are poorly investigated. Whether prenatal exposure to pollutants has an effect on birth weight is incompletely understood. We therefore aimed (1) to determine placental transfer rates of PFAS, BPA, Pb, THg, and MeHg, (2) to analyze relationships between fetal exposure and birth outcome and (3) to analyze gene variants as mediators of placental transfer rates and birth outcome. Two hundred healthy pregnant women and their newborns participated in the study. BPA, 16 PFAS, THg, MeHg, and Pb were determined using HPLCMS/MS (BPA, PFAS), HPLC-CV-ICPMS (MeHg), CV-AFS (THg), and GF-AAS (Pb). Questionnaires and medical records were used to survey exposure sources and birth outcome. 20 single nucleotide polymorphisms and two deletion polymorphisms were determined by real-time PCR from both maternal and newborn blood. Genotype-phenotype associations were analyzed by categorical regression and logistic regression analysis. Specific gene variants were associated with altered placental transfer of PFAS (*ALAD* Lys59Asn, *ABCG2* Gln141Lys), THg (*UGT* Tyr85Asp, *GSTT1*del, *ABCC1* rs246221) and Pb (*GSTP1* Ala114Val). A certain combination of three gene polymorphisms (*ABCC1* rs246221, *GCLM* rs4130*3970, HFE* His63Asp) was over-represented in newborns small for gestational age. 36% of Austrian and 75% of Slovakian mothers had levels exceeding the HBM guidance value I (2 μg/L) of the German HBM Commission for PFOA. 13% of newborns and 39% of women had Ery-Pb levels above 24 μg/kg, an approximation for the BMDL_01_ of 12 μg/L set by the European Food Safety Authority (EFSA). Our findings point to the need to minimize perinatal exposures to protect fetal health, especially those genetically predisposed to increased transplacental exposure.

## Introduction

The objective of the transnational human biomonitoring (HBM) project ‘‘UmMuKi^1^ : chemicals in mothers and their newborns in the Bratislava-Vienna region,” was to compare the exposure situation of pregnant women and its potential effect on birth weight in greater urban areas. Vienna (approximately 1,900,000 inhabitants), Austria, and Bratislava (approximately 420,000 inhabitants), Slovakia, are two Central European cities at approximately 50 km distance.

A strong focus in the UmMuKi study was on Perfluoroalkyl substances (PFAS) that are covered by International Conventions such as the Stockholm Convention on Persistent Organic Pollutants. Other substances of very high concern (SVHC) recognized under the European REACH^2^ Regulation are Bisphenol A (BPA) and lead (Pb). Mercury is individually addressed in the UN Minamata Convention which has entered into force in 2017.

Two PFAS, perfluorooctane acid (PFOA) and perfluorooctansulfonic acid (PFOS) are already restricted within the European Union to minimize exposures. Because the compounds are so persistent, it can be assumed that exposures will decrease slowly. For many other PFAS, the knowledge about their current uses and hazards is still very limited or missing entirely. Recently, 4730 PFAS related substances were identified by OECD^3^. Scientists, regulators, and civil society organizations are calling for effective and efficient assessment and management of PFAS not regulated so far (Ritscher et al., 2018). In addition, PFOS/PFOA are substituted by other PFAS and therefore exposure to these PFAS will increase within the next years and decades.

Previous data indicated low to moderate exposure to Pb and Hg in the Bratislava-Vienna region (e.g., Palkovicova et al., 2008; Gundacker et al., 2010a; Borošová et al., 2014). Exposure to PFAS, however, has not been investigated in this region before 2012. Also for BPA the information was scarce (Hohenblum et al., 2012).

Pregnant women and their newborns constitute a specifically vulnerable population. PFAS, BPA, Pb and Hg are known to be transferred via the placenta to the fetus with the potential to cause placental dysfunction and adversely affect fetal health (Esteban-Vasallo et al., 2012; Gundacker and Hengstschläger, 2012; Troisi et al., 2014; Govarts et al., 2018; Mao et al., 2020). The placental transfer rate, the ratio of umbilical cord to maternal blood concentrations, is a very informative marker of placental toxicokinetics as it indicates the permeability of the placenta to toxicants. Placental transfer rates vary over a wide range within a population as well as between populations (e.g., Stern and Smith, 2003), regardless of the level of exposure. It is reasonable to assume that genetic factors could explain this variability. In genotype-phenotype studies, very often only the maternal genotype is analyzed. From a genetic point of view, a distinction must be made between the contribution of the maternal genotype and the infant genotype to placental transfer capacities. The maternal genes determine the amount of xenobiotics present in maternal blood that come into contact with the placenta. The fetal genotype, however, determines the (transport) phenotype of the placental barrier, since all cell layers involved (syncytiotrophoblast, cytotrophoblast, placental endothelial cells) develop from embryonic cells (Gundacker et al., 2016).

Also the search for genetic variants associated with adverse birth outcomes is ongoing (e.g., Stalman et al., 2018). Small for gestational age (SGA), defined as < 10th growth percentile for gestational week, is among the leading causes of neonatal, infant, and childhood morbidity, mortality, and neurodevelopmental impairment, moreover it can adversely affect health during adult life (e.g., Gillman, 2005). The relationships between prenatal PFAS, BPA, Pb, and Hg exposures and fetal growth are complex and the findings very often inconsistent (Zhu et al., 2010; Nishioka et al., 2014; Murcia et al., 2016; Govarts et al., 2018; Lee et al., 2018; Kamai et al., 2019; Punshon et al., 2019). It was therefore indicated to analyze the potential relationships between adverse birth outcome (i.e., SGA), placental transfer rates of PFAS, BPA, Hg, and Pb, and functional gene sequence variants.

Based on previous studies that were showing certain gene variants to be associated with exposure levels of Hg, Pb, and other toxic metals (Gundacker et al., 2009, 2010b; Kayaalti et al., 2010; Goodrich et al., 2011), we selected a set of common functional polymorphisms in genes encoding for metallothioneins (MTs), Glutathione S-transferases (GSTs), glutamyl-cysteine ligase (GCL), UDP glucuronyltransferases (UGT), Vitamin D receptor (VDR), delta-aminolevulinic acid dehydratase (ALAD), Hemochromatosis (HFE), and ABC transporters (MDR1, MRP1, MRP2, BSEP, BCRP) to detect genotype-phenotype relationships in the perinatal period.

To conclude, the UmMuki study aims were to compare the regional exposure levels and sources of PFAS, BPA, Pb, total Hg, and methyl-Hg (MeHg), to analyze the influence of gene variants on placental transfer rates and birth outcome, and to assess the potential health risks deriving from the exposures to 16 PFAS (PFOS, PFOA, PFNA, PFHxS, PFDA, PFUnDA, PFBA, PFPeA, PFHxA PFHpA, PFDoA, PFTrDA, PFTeDA, PFBS, PFHptS, PFDS), BPA, total Hg (THg), methyl mercury (MeHg), and Pb.

## Materials and Methods

### Study Group

**FIGURE 1:**
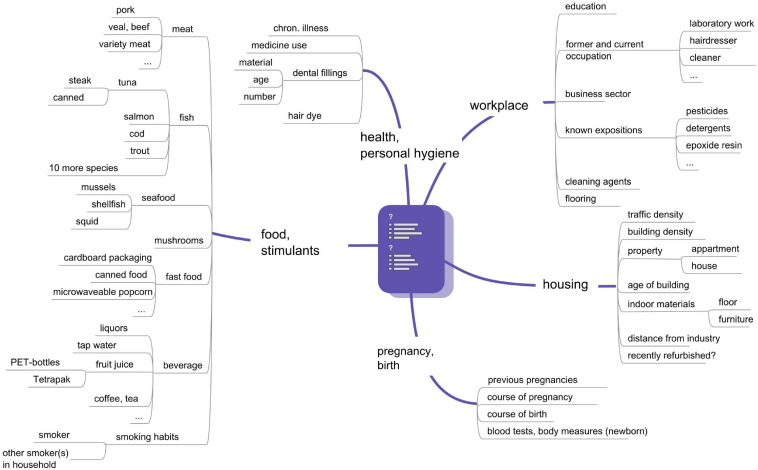
Questionnaire items of the UmMuKi study.

Two hundred and thirty-four healthy pregnant women were recruited at the University clinic in Bratislava and at the Semmelweisklinik in Vienna, respectively, between 2010 and 2012. The study was approved by the ethic committee of the City of Vienna (EK 09-191-1109) and of the University clinic in Bratislava (EK-62-2010). All women, who met the inclusion criteria (i.e., healthy single term pregnancy, age of 18–45 years, signed informed consent), were enrolled in the study. Women having allergies (hay fever and allergic asthma) and hypothyreodism were not excluded. Thirty four women were not eligible because of gestational or sampling complications. Finally, 100 mother-child-pairs in Bratislava and 100 mother-child-pairs in Vienna participated in the study (Table 1). Study participants were interviewed via questionnaires by trained personal at the antenatal units. The recruiting interview was followed by another during gestational week (GW) 36–38, when maternal blood collection took place, and a third within 1–5 days after birth. The questionnaire items are depicted in Figure 1. Clinical data including pregnancy outcome (gestational age, birth weight, birth length, head circumference) were taken from medical records. The classification into small for gestational age (SGA) and large for gestational age (LGA) was made according to the growth percentiles depending on the week of gestation in which birth took place (SGA: birth weight < 10th growth percentile, LGA: Birth weight > 90th percentile). The group in between (10–90th perc.) was designated as AGA (adequate for gestational age).

**TABLE 1:** Characteristics of the study groups in Bratislava and Vienna.

	Bratislava		Vienna		
		
	Mean (range)	N	Mean (range)	N	*p*^*a*^
Pregnant women		100		100	
Age (years)	31 (18–43)		31 (18–43)		ns
Pre-pregnancy BMI	22 (16–32)		22 (16–37)		ns
Pregnancy BMI	28 (20–37)		27 (20–39)		ns
Parity	1.9 (1–7)		1.8 (1–5)		ns
Gestational length (weeks)	40 (37–42)		40 (37–42)		ns
Fish consumption (grams per week)	150 (0–1,000)		254 (0–1,050)		<0.05
Pork consumption (%)	57%		86%		<0.001
No. of dental amalgam fillings	7 (0–16)		3 (0–16)		<0.001
Years of smoking	8 (1–18)		9 (1–23)		ns
Occasional alcohol consumption^b^	19		2		<0.001
Basic education only^c^	27		5		<0.001
Living in old buildings^d^	10		47		<0.001
Residential area^e^	21/16/60		3/23/74		<0.001
Children		100		100	
Birth weight (g)	3,441 (2,370–4,690)		3,405 (2,500–4,808)		ns
Birth length (cm)	50 (46–55)		51 (47–59)		ns
Head circumference (cm)	34 (30–38)		34 (32–37)		ns
Females	43		54		ns

*ns, non-significant (P > 0.05); BMI, body mass index.*

*^a^Result from Kruskal Wallis test or Chi square test.*

*^b^One to two glasses of beer or wine per week.*

*^c^Highest completed level of basic education is elementary school or lower secondary school.*

*^d^Constructed before 1945 (indicates presence of leaded water pipes).*

*^e^Rural/suburban/urban.*

### Sample Collection and Preparation

Blood was sampled with the VACUETTE^®^ Safety collection system. 3 × 7 ml of maternal venous whole blood and mucosal swab samples from the oral cavity, were taken within GW 36–38 of pregnancy. 2–3 × 7 ml of cord blood were collected within 1 h after childbirth. One 7 ml replicate of maternal/cord whole blood was centrifuged for 10 min at 3,000 rpm immediately after collection to separate erythrocytes from blood serum. Newborn whole blood (1 ml) was sampled before postnatal day 5. All samples were stored at –20°C until further treatment. After thawing, 1–1.5 g of erythrocytes were digested with a mixture of 4 ml 69 vol% HNO3 (Roth, supra quality) and 0.75–1 ml 30 vol% H_2_O_2_ (Merck). All samples were digested in Teflon vessels in a microwave oven (mls 1,200 mega, Germany). After cooling, each Teflon vessel was rinsed twice with 2 ml H_2_O, and the digestion solution was transferred into a polyethylene (PE) tube and filled up with millipore water to a volume of 10 ml.

For MeHg analyses, a 0.25 ml aliquot of whole blood samples of women and newborns were weighed into 15 ml PE vials. After addition of 2.5 mL of 0.1% v/v 2-mercaptoethanol in 1 M hydrochloric acid, the samples were extracted for 15 min in an ultrasonic bath. After filtration (0.45 μm membrane filters) the samples were analyzed with High Performance Liquid Chromatography-Cold Vapor-Inductively Coupled Plasma Mass Spectrometry (HPLC-CV-ICPMS).

### Genomic DNA Isolation and Genotyping

DNA of 200 mother-child-pairs was genotyped for 20 single-nucleotide polymorphisms (SNPs) (Table 2) and two deletion polymorphisms (GSTT1, GSTM1) in candidate genes that have been linked to metabolism/detoxification of Hg, Pb, PFOS and PFOA: MT1a, MT4, GCLM, GCLC, GSTT1, GSTM1, UGT, VDR, ALAD, HFE, ABCB1, ABCC1, ABCC2, ABCG2, and ABCB11. All genotype frequencies (besides the a priori dichotomized GSTM1 and GSTT1 data, which could not be tested) were at Hardy Weinberg equilibrium.

**TABLE 2:** Nomenclature of examined polymorphisms and variant allele frequencies in 200 mother-child pairs.

Gene	dbSNP	Position (GRCh38)	Type of variation	VAF_chi_	VAF_mat_	VAF_eu_
*MT1a*	rs11640851	chr16:56639315C>A	Missense (Thr27Asn)	0.70	0.68	0.66
*MT4*	rs11643815	chr16:56568886G>A	Missense (Gly48Asp)	0.10	0.16	0.14
*HFE*	rs1800562	chr6:26092913G>A	Missense (Cys282Tyr)	0.05	0.05	0.06
*HFE*	rs1799945	chr6:26090951C>G	Missense (His63Asp)	0.14	0.12	0.14
*VDR*	rs1544410	chr12:47846052C>T	Intronic	0.41	0.39	0.39
*ALAD*	rs1800435	chr9:113391611C>G	Missense (Lys59Asn)	0.07	0.09	0.08
*GSTP1*	rs1695	chr11:67585218A>G	Missense (Ile105Val)	0.28	0.26	0.33
*GSTP1*	rs1138272	chr11:67586108C>T	Missense (Ala114Val)	0.13	0.10	0.08
*GCLC*	rs17883901	chr6:53545239G>A	Upstream	0.10	0.09	0.08
*GCLM*	rs41303970	chr1:93909753G>A	Upstream	0.22	0.24	0.17
*ABCB1*	rs1045642	chr7:87509329A>G	Synonymous	0.52	0.50	0.47
*ABCB1*	rs1128503	chr7:87550285A>G	Synonymous	0.59	0.57	0.57
*ABCB1*	rs2032582	chr7:87531302A>C	Missense (Ser893Ala)	0.60	0.58	0.55
*ABCB11*	rs2287622	chr2:168973818A>G	Missense (Val444Ala)	0.55	0.55	0.60
*ABCB11*	rs497692	chr2:168932506T>C	synonymous	0.57	0.55	0.54
*ABCC1*	rs246221	chr16:16044465T>C	Synonymous	0.29	0.30	0.31
*ABCC2*	rs717620	chr10:99782821C>T	5’untranslated region	0.18	0.18	0.20
*ABCC2*	rs2273697	chr10:99804058G>A	Missense (Val417Ile)	0.22	0.23	0.20
*ABCG2*	rs2231142	chr4:88131171G>T	Missense (Gln141Lys)	0.14	0.14	0.10
*UGT2B15*	rs1902023	chr4:68670366A>C	Missense (Tyr85Asp)	0.50	0.48	0.48

*VAF_*chi*_, variant allele frequencies in children; VAF_*mat*_, variant allele frequencies in mothers; VAF_*eu*_, variant allele frequencies in European (non-Finnish) population (gnomAD browser v2.1.1).*

Genomic DNA was extracted from maternal buccal swab samples, from frozen maternal peripheral blood and from frozen cord blood using the NucleoSpin Blood DNA extraction kit (Machery-Nagel). Genotyping was performed using commercially available TaqMan Assays (Life Technologies). PCR for each SNP were performed in a single reaction tube for wild-type and variant allele simultaneously in a StepOne Real-Time PCR System (Life Technologies). The 10 μl reaction consisted of ABsolute QPCR ROX Mix (Thermo Fischer), 25 ng of genomic DNA, 500 nM of each primer and of the corresponding pair for the TaqMan probes: 100 nM of the 5°C, followed by 45 cycles of 15 s at 95°C and 1 min at 60°C. Alleles were discriminated with StepOne Software v2.0. GSTM1 and GSTT1 deletions were analyzed using TaqMan gene expression assays (Life Technologies) in the single tube with the primers and 5−/− (homozygous deleted genotype) and + /? (homozygous intact or heterozygous genotype), which implies that the differences between homozygous intact and heterozygous genotype could not be investigated. The MT4a polymorphism was investigated by analysis of the PCR products including the SNP positions in multiplex fashion using standard homogeneous MassEXTENDED and iPLEX protocol. The samples were acquired and analyzed using mass spectrometer MassARRAY Compact Analyzer (Sequenom).

All study participants (*N* = 400) were genotyped. In accordance with the fact that the ancestral allele is not always the major allele (Table 2), we do not use this nomenclature in the following, but use the terms major and minor (or variant) allele. For computation, the genotype data of children and women were dichotomized to codes 1 (homozygous major allele) and 2 (heterozygous and homozygous minor allele). According to the differing methods of genotyping, the GSTM1 and GSTT1 genotypes were coded as 1 (wild type and heterozygous) and 2 (homozygous deleted). We determined 23% of children and 18% of women (GSTT1) and 53% of children and 52% of women (GSTM1) to be homozygous carriers of the deletion polymorphisms. The combined infant-maternal genotypes were coded in two categories (1: both women and newborns are homozygous carriers of the major allele, 2: all other genotypes).

A variant allele frequency score was generated for three gene variants associated with SGA, ranging from homozygous carriers of the major allele (score 1) to a maximum presence of variant alleles (score 12) as shown in Figure 5.

### Analytical Methods

Due to budgetary reasons PFAS, BPA, and MeHg were analyzed in subsets (PFAS: 42 mother-newborn-pairs, BPA and MeHg: 40 mother-newborn-pairs). PFAS, BPA, and MeHg concentrations were analyzed in the accredited HBM laboratory of the Environment Agency Austria. THg and Pb were analyzed in the laboratory at the Medical University of Vienna.

#### Bisphenol A (BPA)

Levels of free BPA in 40 blood samples were measured in 2012 at the accredited HBM laboratory of the Environmental Agency by HPLC-MS. Special efforts have been undertaken to avoid potential artifacts and to prevent false-positive detection of free BPA. Among these Bisphenol A free sampling material was chosen and analyzed previously to detect potential contamination sources. Blood samples were spiked with an isotopically labeled surrogate standard and extracted by SPE. The samples were analyzed with the same LC-MS/MS system as PFAS. For chromatographic separation a Luna C18 100 × 2 mm, 5 μm column (Phenomenex) was used with a gradient formed by water modified with hydrogen carbonate (pH 7) and acetonitrile. The analytical run was 9 min. External calibration was performed on eight levels ranging from 0.5 to 100 ng/mL. Sample extracts were measured once undiluted and once diluted (1/4 v/v). Positive findings were corrected with the recovery rate of the surrogate standard.

#### PFAS

The levels of 19 PFAS were measured in randomly selected maternal and cord blood serum samples after preparation by solid phase extraction (SPE) using an Agilent Technologies 1290 Infinity Series (Agilent Technologies, Santa Clara, CA, United States) as HPLC system and an AB Applied Biosystems MDS SCIEX 4000 QTRAP LC/MS/MS System (AB Sciex Technologies, Framingham, MA, United States) as MS detector system. The detection was performed through specific mass transitions in electrospray ionization (ESI) negative mode and the quantification in multiple reaction monitoring (MRM) mode. Two analytical columns were used to ensure reliable separation of the target analyte peaks from the matrix (Luna C18 100 × 2 mm, 5 μm, and Kinetex PFP, 100 × 2.1 mm, 2,6 μm, both from Phenomenex, Aschaffenburg, Germany). Injection volume in both cases was 10 μL. The separation of measured PFAS was conducted by a gradient elution method using water modified with 10 mM ammonium acetate and methanol (Luna) or acetonitrile (Kinetex). Analytical runs were 25 min. and 60 min, respectively. External calibration was performed on 13 levels ranging from 0.1 to 25 ng/mL. Each sample was measured once with each chromatographic method. Analytical results are given as their means corrected by the blanks and recoveries of the corresponding internal standard. The limits of quantification (LOQs) were determined according to DIN 32645 (DIN 2008).

#### Pb and Total Mercury (THg)

There was insufficient volume (<0.5 ml) of some blood samples to process them further, which explains the slightly lower number of samples for CordEry-THg, MatEry-THg, and MatEry-Pb (Table 3). Pb concentrations were analyzed by graphite furnace technique using the Hitachi Z-8200 AAS. Analyses of THg were conducted by using the mercur plus CV-AFS (Analytik-Jena, Germany). Quality control was assured by measuring blank test solutions and reference materials at each analysis run (Seronorm Trace Elements Human Whole Blood L-2, 210205). THg concentration of reference material (LOT 1003129: 16.1 ± 1.3 μg/L) and Pb content of reference material (LOT 1003192: 338 ± 21 μg/L) were in accordance with the certified levels for THg (16.0 ± 3.2 μg/L) and Pb (336 ± 18 μg/L). The limit of detection (LOD) was 0.07 μg/L (THg) and 1.7 μg/L (Pb). All metal contents were measured in duplicate (RSD < 10%) by the working curve method.

**TABLE 3:** Concentrations of Bisphenol A (BPA), Perfluoralkyl substances (PFAS), total mercury (THg), methyl mercury (MeHg), and lead (Pb) in maternal and newborn blood samples.

	N	AM ± SD	90th Perc.	MAX	N < LOD
CordS-BPA (μg/L)	42	0.3 ± 0.7	1.2	3.8	29(73%)
MatS-BPA (μg/L	42	0.5 ± 0.9	1.7	3.5	26(65%)
CordS-PFAS^a^ (μg/L)	42	2.9 ± 1.7	5.3	7.2	0(0%)
MatS-PFAS^a^ (μg/L)	42	6.5 ± 4.5	13.1	23.1	0(0%)
CordS-PFOA (μg/L)	42	2.0 ± 1.4	4.2	5.8	1(2%)
MatS-PFOA (μg/L)	42	3.5 ± 3.6	8.6	16.5	1(2%)
CordS-PFOS (μg/L)	42	0.5 ± 0.5	1.2	2.5	9(21%)
MatS-PFOS (μg/L)	42	1.7 ± 1.0	3.2	3.8	3(7%)
CordS-PFNA (μg/L)	42	0.2 ± 0.2	4.4	1.0	5(12%)
MatS-PFNA (μg/L)	42	0.5 ± 0.3	0.9	1.7	3(7%)
CordS-PFHxS(μg/L)	42	0.08 ± 0.16	0.2	0.9	25(60%)
MatS-PFHxS (μg/L)	42	0.3 ± 0.2	0.6	1.3	8(19%)
CordS-PFDA (μg/L)	42	0.04 ± 0.06	1.0	0.3	24(57%)
MatS-PFDA (μg/L)	42	0.2 ± 0.2	0.5	1.1	3(7%)
CordS-PFUnDA (μg/L)	42	0.02 ± 0.07	0.1	0.4	35(83%)
MatS-PFUnDA (μg/L)	42	0.2 ± 0.2	0.6	1.0	12(29%)
CordEry-THg (μg/kg)	189	2.6 ± 1.5	4.1	11.4	0(0%)
MatEry-THg (μg/kg)	182	1.8 ± 1.1	3.3	8.1	0(0%)
ChildB-MeHg (μg/L)	40	1.5 ± 1.8	4.5	8.4	2(5%)
MatB-MeHg (μg/L)	40	0.9 ± 1.0	2.8	4.2	8(20%)
CordEry-Pb (μg/kg)	200	16 ± 21	27	182	0(0%)
MatEry-Pb (μg/kg)	198	27 ± 27	51	234	0(0%)

*AM ± SD, arithmetic mean ± standard deviation; Perc, Percentile; MAX, maximum value; < LOD, below limit of detection; CordS, umbilical cord serum; MatS, maternal serum; CordEry, umbilical cord erythrocytes; MatEry, maternal erythrocytes; CordB, cord whole blood; MatB, maternal whole blood.*

*^a^Sum PFAS (PFOS, PFOA, PFNA, PFHxS, PFDA, PFUnDA, PFBA, PFPeA, PFHxA PFHpA, PFDoA, PFTrDA, PFTeDA, PFBS, PFHptS, PFDS).*

#### Methyl Mercury (MeHg)

A subsample of 80 whole blood specimen (20 mother-child pairs in Bratislava and Vienna, respectively) was analyzed for MeHg. The selection was made on the basis of THg levels, i.e., the twenty samples with highest (>80th percentile) and lowest (<20th percentile) concentrations were chosen. We analyzed MeHg by HPLC-CV-ICPMS with a mobile phase (60 mM ammonium acetate, 0.1% v/v mercaptoethanol, 5% v/v methanol at pH 6.8), within 5 min on Atlantis dC18 (4.6 × 20 mm) chromatographic guard column at 20°C. Post column reduction was performed with 1% w/w sodium borohydride and 1 M hydrochloric acid followed by ICPMS detection at m/z 202. For quality assurance, analysis of extraction blanks, reference material NIST 955c-3 (Toxic metals in caprine blood), sample duplicates and spiked samples was performed. Recovery of MeHg for reference material NIST 955c-3 was 98 ± 7% (*N* = 4) and for spiked samples 95 ± 5% (*N* = 4). The relative standard deviation of duplicate samples was 4 ± 2.5% (*N* = 7). The LOQ and LOD for MeHg were 0.7 μg/L, respectively, 0.1 μg/L. MeHg concentrations < LOQ were replaced with the half LOQ.

### Statistics

Placental transfer rates were calculated by dividing the cord blood/serum concentrations by the maternal blood/serum concentrations for BPA, sumPFAS, PFOA, PFOS, PFNA, PFDA, PFUnDA, PFHxS, THg, MeHg, and Pb (Figure 3A). A ratio of 1 means equal concentrations in maternal blood and cord blood.

Bivariate associations between questionnaire data and medical record data with placental transfer rates, birth outcome (SGA vs. AGA), and genotypes were determined. Data from LGA babies (as *N* < 5) were excluded from these analyses. Non-parametric tests were used for placental transfer rates of BPA, PFAS, THg, MeHg, and Pb. Group differences and quota were analyzed with Mann-Whitney test, Kruskal-Wallis test and Chi-Square test. Spearman correlation was used in correlation analyses. In order to not increase the risk for false-negative findings, we applied *P* < 0.1 (and close to it) as the inclusion criteria into the further multivariate statistics using categorical regression analysis (CATREG) for metric outcome variables (placental transfer rates) and logistic regression for the nominal variable birth outcome (SGA-AGA).

All variables associated with placental transfer rates in bivariate analyses (*P* ≤ 0.11) were included into CATREG models (crude models). The CATREG analysis provides the Pratt-coefficient, which ranks the independent variables to their relative importance within a model. The non-significant and/or unimportant factors in this order were stepwise eliminated from the regression model using *P* > 0.1 and Pratt-Coefficient < 0.05 as elimination criteria (final models). For numerical predictors of birth weight, their significance and importance for binary outcomes (SGA, AGA) were assessed by their logistic regression coefficients. For this purpose, predictors were removed stepwise from the full model (containing all predictors) until further removal significantly lowered the prediction quality (AIC). Statistics were calculated with IBM SPSS Version 26 and R 4.0.0. The tests were performed as two-sided and *P* < 0.05 was set as the significance level.

## Results

### BPA, PFAS, THg, MeHg, and Pb Concentrations of Mother-Child-Pairs

As Table 1 shows, the study groups in Bratislava and Vienna differed in terms of eating habits, education and housing. Only some of these factors can explain differences in exposure to PFAS, Hg and Pb as outlined below.

**FIGURE 2:**
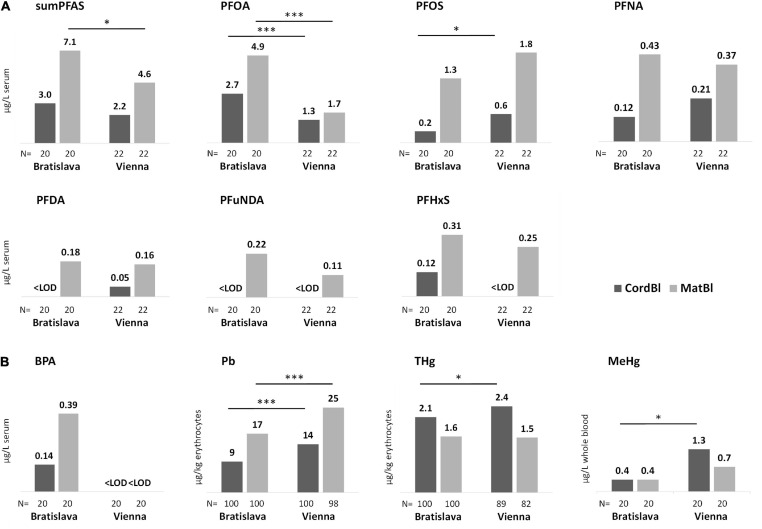
Median levels of **(A)** PFAS and **(B)** BPA, Pb, THg and MeHg in cord blood and maternal blood specimens. Site-specific differences are marked with asterisks. Kruskal Wallis-test *P < 0.05 and ***P < 0.001.

BPA was detected in 27% of cord serum and 35% of maternal serum samples (Table 3), mostly from Bratislava (Figure 2B). BPA levels of women and newborns were significantly associated (*r* = 0.548, *P* < 0.001). In this low-exposed subgroup no correlation was found with known exposure factors such as consumption of canned food, occurrence or removal of dental inlays of synthetic origin, floors at home (e.g., PVC or laminate flooring). BPA data were excluded from all further statistical tests because of their limited information.

PFOA and PFOS contributed most to the PFAS content in serum (Table 3) with some significant differences in exposure between Bratislava and Vienna (Figure 2A). Site-specific sources of exposure could not be identified. PFAS concentrations in women and newborns correlated significantly (Supplementary Table 1). In CATREG analyses, MatS-PFAS concentrations and gestational length were significant predictors of CordS-PFAS concentrations. The main modulators of MatS-PFAS concentrations were study site and parity (Supplementary Table 2).

THg and MeHg as well as Pb blood levels of women and newborns correlated well (Supplementary Table 3). Significantly higher Pb concentrations were observed for study participants from Vienna in comparison to those in Bratislava. This was also true for THg and MeHg with regard to the blood levels of newborns (Figure 2B).

Fish consumption and maternal age were significant predictors of CordBl-THg, while fish consumption and number of amalgam fillings were the significant factors determining MatEry-THg (Supplementary Table 5). We observed higher cord blood mercury levels in boys than in girls (2.4 vs. 1.9 μg/kg; *P* = 0.035) that, however, were not attributable to enhanced fish consumption by women giving birth to boys (data not shown). The significant determinants of maternal Pb levels were study site, age of residential building, i.e., living in buildings constructed before or after 1945, and maternal age (Supplementary Table 5).

### Placental Transfer of BPA, PFAS, THg, MeHg, and Pb

**FIGURE 3:**
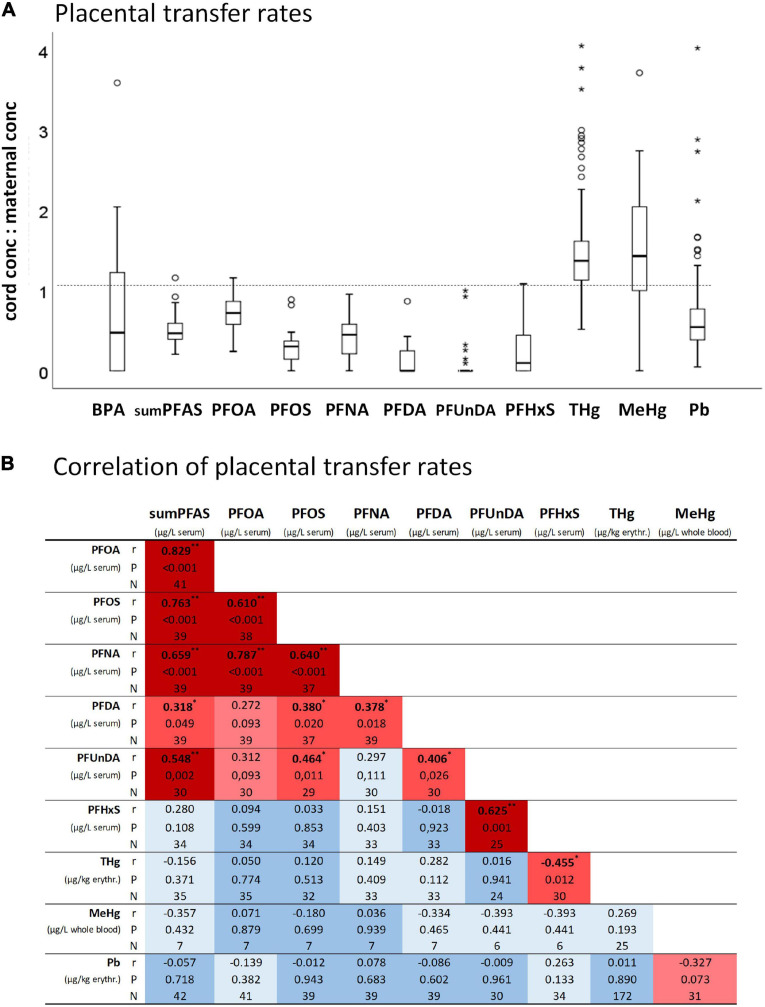
**(A)** Placental transfer rates (i.e., umbilical cord blood concentrations to maternal blood concentrations) of BPA (*N* = 14), sumPFAS (*N* = 42), PFOA (*N* = 41), PFOS (*N* = 39), PFNA (*N* = 39), PFDA (*N* = 39), PFUnDA (*N* = 30), PFHxS (*N* = 34), THg (*N* = 172), MeHg (*N* = 32), Pb (*N* = 198). **(B)** Correlation heatmap of placental transfer rates. *P < 0.05 and **P < 0.01 from Spearman correlation analyses.

Placental transfer rates were toxicant-specific in the following order: MeHg > THg > PFOA > Pb > BPA > sumPFAS > PFNA > PFOS > PFHxS > PFDA > PFUnDA (Figure 3A). Most transfer rates of PFAS are significantly correlated (Figure 3B). An exception is PFHxS, whose transfer rate -within the PFAS–only correlates with that of PFUnDA. The correlation matrix also shows that the transfer rates of PFAS, Pb, THg and MeHg are, with few exceptions, not related to each other.

In multivariate analysis, maternal education, and two gene variants (ALAD: rs1800435; ABCG2: rs2231142) when present in both mother and child, remained the most significant predictors of placental PFAS transfer (Tables 4, 5). Placental transfer of THg was reduced when pregnant women had amalgam fillings, ate pork meat and carried the GSTT1 deletion polymorphism. The infant genetic variants that modulate THg transfer to the fetus are UGT2B15 (rs1902023) and ABCC1 (rs246221) (Table 6). Maternal education, maternal smoking habits and a common GSTP1 polymorphism (rs1138272), when the mother carried the gene variant, remained significant predictors of placental Pb transfer (Table 7).

**TABLE 4:** Genotypes associated with placental transfer rates (PlTR) in bivariate statistics.

Gene polymorphism	Alleles	N	PlTR sumPFAS^a^ (range)	*P*^§^
*ALAD* (rs1800435)_comb	CG/GG, CG/CG, CG/CC^b^	7	0.41 (0.31–0.45)	0.041
(G > C, Lys59Asn)	GG/GG	35	0.51 (0.21–1.16)	
*ABCG2* (rs2231142)_comb	CC/CC	31	0.45 (0.21–0.92)	0.053
(C > A, Gln141Lys)	CA/CC, CA/CA, CA/AA, AA/AA	11	0.51 (0.40–1.16)	

			**PlTR THg (range)**	

*GSTT1* deletion_mat	del/+, +/+	138	1.33 (0.52–3.78)	0.057
	del/del	34	1.51 (0.85–4.05)	
*UGT2B15* (rs1902023)_inf	TT	43	1.25 (0.63–4.05)	0.027
(T > G, Tyr85Asp)	GT, GG	129	1.41 (0.52–3.78)	
*ABCC1* (rs246221)_inf	TT	93	1.32 (0.52–4.05)	0.057
(T > C, Val275 =)	CT, CC	79	1.45 (0.62–3.78)	

			**PlTR Pb (range)**	

*GSTP1* (rs1138272)_mat	CT, TT	24	0.44 (0.24–1.03)	0.032
(C > T, Ala114Val)	CC	174	0.56 (0.05–5.68)	

*Genotypes arranged with increasing median placental transfer rates.*

*^a^sumPFAS: sum parameter of PFOS, PFOA, PFNA, PFHxS, PFDA, PFUnDA, PFBA, PFPeA, PFHxA PFHpA, PFDoA, PFTrDA, PFTeDA, PFBS, PFHptS, PFDS concentrations.*

*^b^CC/CC combination was not present as just one woman was homozygous for CC.*

*^§^ Kruskal Wallis test P for different placental transfer rates according to genotype. Comb, combined maternal-infant genotype; mat, maternal genotype, inf, infant genotype.*

**TABLE 5:** Factors associated with placental transfer rate of sumPFAS^§^ (CATREG model).

Factors	β± S.E^a^	*P*	Partial r [R^2^]	Importance coeff. (rank)
Maternal education	0.368 ± 0.117	<0.001	0.432	0.393 (1)
ALAD (rs1800435)_ comb	0.379 ± 0.142	0.011	0.429	0.354 (2)
ABCG2 (rs2231142)_ comb	0.344 ± 0.136	0.015	0.398	0.253 (3)
[*Crudemodelwith* 5*factors*]^b^		<0.001	[0.504]	
[*Finalmodelwith* 3*factors*]		<0.001	[0.388]	

*^a^ Standardized slope ± standard error.*

*^b^ The crude model included five factors associated with placental PFAS transfer in bivariate tests, i.e., gestational length (d), maternal hemoglobin (g/dl), maternal education (highest completed education was coded 1: grammar school, 2: higher education college, master profession, 3: university), ALAD (rs1800435)_ combined maternal-infant genotype (coded: 1 = GG, 2: CG, CC), ABCG2 (rs2231142)_ combined maternal-infant genotype (coded: 1 = GG, 2: Gt, TT).*

*^§^PFAS: sum parameter of PFOS, PFOA, PFNA, PFHxS, PFDA, PFUnDA, PFBA, PFPeA, PFHxA PFHpA, PFDoA, PFTrDA, PFTeDA, PFBS, PFHptS, PFDS concentrations. comb, combined maternal-infant genotype.*

**TABLE 6:** Factors associated with placental transfer rate of THg (CATREG model).

Factors	β± S.E.^a^	*P*	Partial r [R^2^]	Importance coeff. (rank)
No of maternal amalgam fillings	−0.462 ± 0.070	<0.001	−0.488	0.695 (1)
*UGT2B15* (rs1902023)_inf	0.172 ± 0.061	<0.001	0.206	0.087 (2)
Consumption of pork	−0.143 ± 0.063	0.007	−0.170	0.083 (3)
*GSTT1* (del)_mat	0.156 ± 0.060	0.010	0.187	0.070 (4)
*ABCC1* (rs246221)_inf	0.142 ± 0.066	0.032	0.171	0.065 (5)
[*Crudemodelwith* 11*factors*]^b^		<0.001	[0.453]	
[*Finalmodelwith* 5*factors*]		<0.001	[0.341]	

*^a^Standardized slope ± standard error.*

*^b^The crude model included 11 factors associated with placental PFAS transfer in bivariate tests, i.e., study site (1 = Vienna, 2 = Bratislava), maternal age (yrs), maternal years of smoking, acute illness (1 = no, 2 = yes), maternal education (highest completed education was coded 1: grammar school, 2: higher education college, master profession, 3: university), number of amalgam fillings, consumption of pork (1 = never, 2 = 1–2 times per week, 3 = 3–7 times per week, 4 = more than 7 times per week), GSTM1 (del)_maternal genotype, GSTT1 (del)_maternal genotype, ABCC1 (rs246221) infant genotype, UGT2B15 (rs1902023)_infant genotype.*

*inf, infant genotype; mat, maternal genotype.*

**TABLE 7:** Factors associated with placental transfer rate of Pb (CATREG model).

Factors^*a*^	β± S.E^a^	*P*	Partial r [R^2^]	Importance coeff. (rank)
Maternal education	0.368 ± 0.117	<0.001	0.432	0.393 (1)
*GSTP1* (rs1138272)_mat	0.379 ± 0.142	0.011	0.429	0.354 (2)
Maternal years of smoking	0.344 ± 0.136	0.015	0.398	0.253 (3)
[*Crudemodelwith* 5*factors*]^b^		<0.001	[0.504]	
[*Finalmodelwith* 3*factors*]		<0.001	[0.388]	

*^a^Standardized slope ± standard error.*

*^b^The crude model included five factors associated with placental Pb transfer in bivariate tests, i.e., maternal age (yrs), maternal years of smoking, maternal education (highest completed education was coded 1: grammar school, 2: higher education college, master profession, 3: university), MT1a_maternal genotype, GSTP1_maternal genotype.*

*mat, maternal genotype.*

### Birth Outcome

**FIGURE 4:**
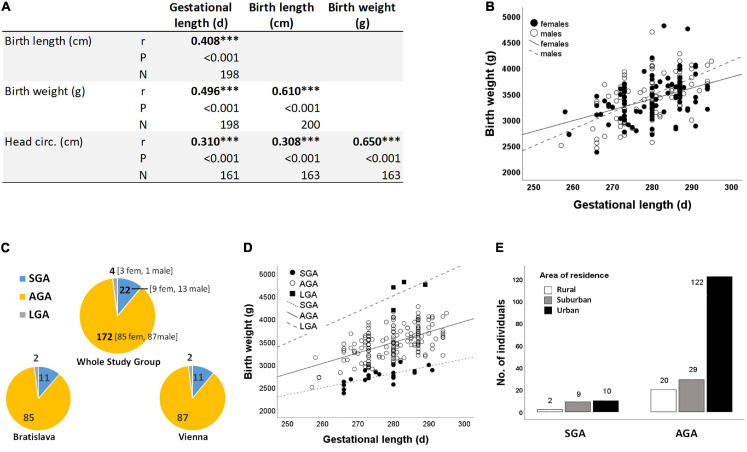
**(A)** Correlations between neonatal anthropometry and gestational length ***P < 0.001 (Spearman correlation) **(B)** as exemplified for birth weight and gestational length in subgroups of girls (*r* = 0.583, *N* = 97) and boys (*r* = 0.417, *N* = 103). **(C)** Birth outcome (i.e., SGA, AGA, and LGA) is almost the same at both study sites (Chi-square test *P* = 0.998). **(D)** Birth weight increases with gestational length independent of birth outcome (SGA: *r* = 0.711, *N* = 22; AGA: *r* = 0.551, *N* = 172; LGA: *r* = 0.515, *N* = 4). **(E)** Relationship between residential area and birth outcome (Chi-Square test *P* = 0.019). Three study participants did not provide information on residential area (Table 1), resulting in smaller numbers of SGA cases (*N* = 21) and AGA cases (*N* = 171) here.

Birth weight, birth length and head circumference were significantly associated with gestational length (Figure 4A), with boys being more likely to benefit from a longer gestation period than girls (Figure 4B). 11% of newborns were SGA at each study site (Figure 4C). Although males were numerically overrepresented in the SGA group (Figure 4C), there was no statistically significant difference between neonatal sex and birth outcome (Chi-Square test *P* = 0.434).

**FIGURE 5:**
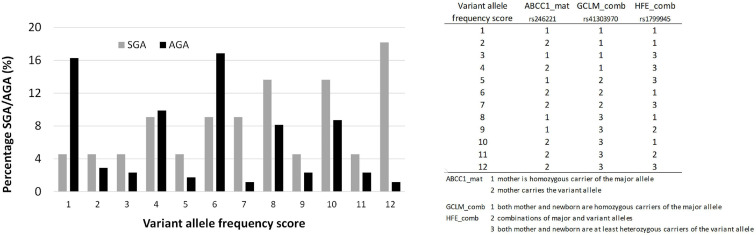
The distribution of *ABCC1*, *GCLM*, and *HFE* alleles in AGA and SGA cases. Chi-Square test *P* < 0.05.

Birth weight always increased with gestational length independent of birth outcome (Figure 4D). The further factors associated with birth outcome (SGA-AGA) in bivariate statistics were: residential area (Figure 4E), and five gene variants related to maternal genotype (ABCC1, rs246221), infant genotype (VDR, rs1544410) or the combined maternal and infant genotypes (GCLM, rs41303970; HFE, rs1799945; ABCC2, rs717620) (Table 8). The logistic regression confirmed these genotypes to be associated with birth outcome (Table 9), of which ABCC1, GCLM, and HFE variant genotypes were over-represented in the SGA group when mothers and/or children carried the variant alleles; vice versa AGA newborns were more often homozygous for the major allele (Figure 5). A VDR variant genotype (rs1544410), gestational length and residential density (i.e., living in urban areas) were significantly associated with AGA (Table 9).

**TABLE 8:** Genotypes associated with birth outcome in bivariate analysis.

Gene polymorphism	Alleles and allele combinations	SGA (N)	AGA (N)	*P*^§^
*VDR* (rs1544410)_inf	GG	14	56	0.008
(G > A, intronic)	GA, AA	8	116	
*ABCC1* (rs246221)_mat	TT	6	87	0.044
(T > C, synonymous)	CT, CC	16	85	
*HFE* (rs1799945)_comb	CC/CC	11	116	0.105
(C > G, His63Asp)	CC/CG, CG/CG, CG/GG, GG/GG	11	56	
*GCLM* (rs41303970)_comb	CC/CC	6	83	0.063
(C > T, upstream)	CC/CT, CT/CT, CT/TT, TT/TT	16	89	
*ABCC2* (rs717620)_comb	CC/CC	16	89	0.063
(C > T, 5′ untranslated)	CT/CC, CT/CT, CT/TT, TT/TT	6	83	

*^§^ Chi-square test P for genotype frequencies (SGA vs. AGA).*

*SGA, small-for-gestational-age; AGA, adequate-for-gestational-age.*

*inf, infant genotype comb: mat, maternal genotype; comb, combined maternal-infant genotype.*

**TABLE 9:** Factors associated with SGA (Log-linear model).

	Estimate	Std. error	*z*-value	Pr(>| z|)	Importance (rank)
(Intercept)	–3.1292	0.4542	–6.889	5.62e-12***	
*ABCC1* (rs246221)_mat	0.9804	0.2978	3.292	0.000993***	3.292447 (1)
*VDR* (rs1544410)_inf	–0.8835	0.3205	–2.757	0.005837**	2.756785 (2)
Gestational length (d)	–0.8192	0.2981	–2.748	0.006000**	2.747798 (3)
Residential area^§^	–0.7778	0.2932	–2.653	0.007987**	2.652601 (4)
*GCLM* (rs41303970)_comb	0.7294	0.2857	2.553	0.010690*	2.552679 (5)
*HFE63* (rs 1799945)_comb	0.5253	0.2433	2.159	0.030880*	2.158609 (6)
*ABCC2* (rs717620)_comb	–0.5050	0.2940	–1.718	0.085886	1.717508 (7)

***P < 0.001, **P < 0.01, *P < 0.05.

*^§^ coded 1, rural; 2 (, *suburban; 3*(, *urban.**

*mat, maternal genotype; inf, infant genotype; comb, combined infant-maternal genotype.*

We did not find the birth outcome associated with PFAS, THg, MeHg and Pb levels of mother-newborn-pairs nor with placental transfer rates (*P* > 0.05, respectively). Regarding neonatal anthropometry, weak but statistically significant associations (*P* < 0.05) were observed between CordBl-Hg and birth length (*r* = 0.152, *N* = 189) and between CordBl-Pb and head circumference (*r* = 0.154, *N* = 163). Several PFAS showed a significant positive correlation with gestational length (CordS-sumPFAS: *r* = 0.332; CordS-PFOA: *r* = 0.306; CordS-PFHxS: *r* = 0.342; *P* < 0.05, *N* = 42, respectively); this was the case also for placental transfer of PFNA (*r* = 0.355, *P* = 0.026, *N* = 39).

## Discussion

### Exposure to BPA, PFAS, THg, MeHg, and Pb

BPA is listed as substance of very high concern for reproductive toxicity (classified as reprotoxic chemical of the category 1B, CLP) and as endocrine disrupter for human health by the European Chemicals Agency ECHA^4^. BPA exposure of Austrian women and newborns is comparably low, which is confirmed by a previous Austrian study, where 16% of samples had BPA urine levels in a detectable range (Hohenblum et al., 2012); this, however, at a quite high LOQ of 0.6 μg/L. We have no explanation for the higher BPA concentrations in mother-child pairs from Bratislava. Known exposure pathways such as consumption of canned food, dental inlays of synthetic origin, flooring at home (e.g., PVC or laminate flooring) had no influence on BPA levels in this population.

PFAS levels particularly PFOA and PFOS differ substantially between Bratislava and Vienna. The sources for the site-specific exposures, however, are yet unclear. Maternal blood PFOA levels were elevated in Bratislava, not only compared to Vienna but also with regard to other studies (EFSA, 2018). Thirty six percent of Austrian and 75% of Slovakian mothers had levels exceeding the HBM guidance value I (2 μg/L) of the German HBM Commission for PFOA (Apel et al., 2017). In the updated scientific opinion (EFSA, 2020) with a PFAS mixture risk assessment, a lowest BMDL_10_ of 17.5 μg/L was derived for the sum of PFOA, PFNA, PFHxS and PFOS, based on the inverse association between serum levels of the sum of these four PFAS in 1-year-old children and their antibody titres against diphtheria. According to the European Food Safety Authority (EFSA) assessment, this BMDL_10_ in 1-year-old children corresponds to a maternal serum level of 6.9 ng/mL at a maternal age of 35 years and assuming 12 months of breastfeeding. Accordingly, maternal serum levels < 6.9 μg/L shall protect breast-fed children. In the present study, 31% of the women had serum levels > 6.9 μg/L for exposure to the sum of the four PFAS; the maximum sum value was 21.6 μg/L.

Pregnant women in Vienna ate more fish during pregnancy compared to women in Bratislava, which well explains their higher THg levels. With single exceptions the MeHg levels of newborn blood lay below 5 μg/L, the HBM-I value (alert level) set by the German HBM commission for total mercury (Schulz et al., 2012). None of the samples exceeded the HBM-II concentration of 15 μg/L (intervention level). MeHg, however, clearly contributes to THg levels in women and newborns (Table 3), which is in line with previous findings (e.g., Ask et al., 2002).

The German HBM commission suspended the HBM values in 2009 for Pb for several reasons, among them the adverse effects on neurodevelopment of children at blood levels below 100 μg/L and the possibility that Pb-induced effects persist into adulthood (Wilhelm et al., 2010). Similarly, the European Food Safety Authority (EFSA, 2010) says there is no threshold for critical Pb-induced health effects. Since 2012 the US CDC^5^ recommends the use of a reference blood Pb level of 50 μg/L to identify children, who require case management. In the UmMuKi study, the maximum CordEry-Pb concentration was 182 μg/kg (which is about 90 μg/L in whole blood). Five women and three newborns from Vienna had blood Pb levels above 100 μg/kg (about 50 μg/L) and 13% of newborns and 39% of women had levels beyond 24 μg/kg, a proxy for the EFSA BMDL_01_ of 12 μg/L based on developmental neurotoxicity (EFSA, 2012). A likely cause for the higher Pb exposure in Vienna is the large fraction of participants living in residential buildings constructed before 1945 (47% in Vienna vs. 10% in Bratislava). Such old buildings are still equipped with legacy lead plumbing, and tap water is a comparatively popular drinking water source in Vienna. The dissemination of effective countermeasures (e.g., draining the nightly stagnation water) would help mitigating lead exposure from tap water. The study site explains most of the variance in the models on Pb exposures (Supplementary Table 5). This implies that there are further so far unidentified sources of Pb exposure in the Bratislava-Vienna region.

### Placental Transfer Rates of BPA, PFAS, THg, MeHg, and Pb

PFAS, BPA and Pb concentrations were consistently lower in cord blood than in maternal blood indicating low transfer capacity of the human placenta for these substances. Overall, the correlation matrix on placental transfer rates speaks for different transfer mechanisms of THg and MeHg in comparison to Pb and PFAS. In-vitro findings from placental cell models showing that L-type amino acid transporters are involved in MeHg uptake (Straka et al., 2016; Balthasar et al., 2017) and that MRP1 is mediating its efflux (Granitzer et al., 2020) may explain why MeHg is transported so efficiently across the human placenta into fetal blood. However, our overall knowledge of the mechanisms responsible for toxicant-specific uptake, metabolism and export at the placental barrier, in particular of PFAS and Pb, is poor.

#### PFAS

We found maternal education and two missense mutations associated with placental PFAS transfer rates. Apart from the general relationship between maternal education and neonatal health (Mensch et al., 2019), it remains unclear what aspect of maternal education—a surrogate variable that indicates socioeconomic, health and nutritional status—can contribute to placental transfer of PFAS. The amino acid substitution in the *ALAD* gene (Lys59Asn) leads to a more electronegative charge of the variant enzyme, which could alter binding affinities (Onalaja and Claudio, 2000). The missense mutation of *ABCG2* (Gln141Lys) is associated with reduced ABCG2 expression (Slomka et al., 2020). The placental passage of PFOS and PFOA, however, has been shown to not depend on ABCG2 in a human placental perfusion model (Kummu et al., 2015).

#### THg

Placental transfer of THg was reduced by the number of maternal amalgam fillings as well as the maternal consumption of pork. Amalgam fillings release inorganic mercury (IHg) (Clarkson, 2002). In endothelial cells treated with IHg or MeHg in the same concentrations, it was shown that IHg (HgII) is taken up into the cytoplasm to higher amounts than MeHg (Liu et al., 2020). In a study from China, pork meat was found to contain much more IHg than MeHg (Li et al., 2010). To our knowledge it has not been shown yet whether IHg prevents the uptake of MeHg into (placental) cells.

UGT2B15 is a drug-metabolizing phase II UDP glucuronosyltransferase (UGT) that metabolizes steroid hormones and is expressed in the human placenta (Lévesque et al., 1997). The missense mutation Tyr85Asp has been shown to be associated with drug clearance (e.g., of oxazepam; He et al., 2009), kinetics of androstane-3alpha, 17beta-diol and dihydrotestosterone (Lévesque et al., 1997), and estradiol levels in female breast cancer patients (Sparks et al., 2004). Hg is a well-known endocrine disruptor (e.g., Tan et al., 2009), however, it remains speculative how the investigated UGT2B15 genotype of infants might have changed placental Hg transfer.

The GST enzyme family protects cells from oxidative stress and toxic chemicals by detoxifying electrophilic compounds. The homozygous GSTT1 deletion, a well-studied polymorphism, impairs the enzyme’s catalytic activity, which increases sensitivity to oxidative stress and toxic substances (Hayes et al., 2005). The GSTT1 homozygous deletion was found associated with decreased urine THg (Goodrich et al., 2011) and higher levels of THg in hair (Gundacker et al., 2007). In the present study and other studies (Custodio et al., 2004, 2005; Lee et al., 2010; Barcelos et al., 2013, 2015), GSTT1 deletion had no effect on blood levels of THg or MeHg. We observed, however, a higher rate of transmission of THg via the placenta when the mother carried the GSTT1 deletion indicating an increased risk of fetal Hg exposure. Further research is necessary to determine the effect of cellular GSTT1 depletion on placental Hg kinetics.

MRP1 (ABCC1) is a multitasking ABC transporter, one of its central functions being to maintain the cellular redox status by the efflux of glutathione (GSH) conjugates (GS-X) and oxidized GSH (GS-SG) (Cole, 2014). Although ABCC1 gene variants were shown to be associated with Hg toxicokinetics (Llop et al., 2014; Engström et al., 2016), it has not yet been clarified whether Hg is transported by MRP1. In a recent work we confirmed Hg as a MRP1 substrate as well as that MRP1 function is essential for the viability of placental cells exposed to MeHg (Granitzer et al., 2020). In the present study, a non-synonymous ABCC1 SNP (rs246221), i.e., the presence of the C-allele in infants, was associated with a higher placental transfer rate of THg. Recent data that we gained in another study group confirm this association (unpublished data Gundacker). To our knowledge, however, it has not yet been researched how this nsSNP affects MRP1 expression and function. The SNP also could be linked to other functional gene variants not present in our panel.

#### Pb

Maternal education and maternal smoking were associated with enhanced Pb transfer across the human placenta. It is known that tobacco smoking deregulates ion transport and expression of antioxidant enzymes and metallothionein in the human placenta (Ronco et al., 2006; Votavova et al., 2011). In which way smoking habits might affect transport of Pb toward fetal blood remains unknown. GSTP1, a cytosolic GST, is highly expressed in the human placenta (Raijmakers et al., 2001). Pb exposure can induce GSTP1 activity in rats (Wright et al., 1998). A common missense polymorphism (Ala114Val) modifies the shape of the active site and thus substrate affinity, resulting in lower catalytic efficiency of GSTP1 (Zhong et al., 2006; Goodrich and Basu, 2012). The variant, however, has been rarely investigated with regard to Pb exposure levels and if so, no associations have been found (Gundacker et al., 2009). Also in the present study, the minor genotype 114Val was not associated with Pb levels of mother-newborn-pairs but with lowered Pb transfer across the placenta when the mother carried the T allele. This may indicate a crucial role of GSTP1 in Pb transfer across the placenta as Pb is likely to be transported by MRPs as a GSH conjugate (Ballatori et al., 2009). The formation of the Pb-GSH conjugate requires the presence of a GST catalyzing this conjugation reaction.

### Birth Outcome

The here observed positive correlations between gestational length and sumPFAS, PFOA, PFHxS levels indicate accumulation with gestational age. PFOS, PFOA, and PFNA were shown to accumulate in placenta across gestation (Mamsen et al., 2019), which may harm placental functions (Szilagyi et al., 2020). A pooled analysis of 7 European birth cohorts found that prenatal exposure to PFOA (median cord serum level: 0.55 μg/L) may contribute to the prevalence of SGA (Govarts et al., 2018).

In the present study, gestational length was associated with birth outcome (SGA, AGA, LGA) confirming gestational age as a strong contributor to birth weight (Clausson et al., 2000; Donahue et al., 2010). Furthermore, AGA babies were over-represented in urban areas of Bratislava and Vienna. Although rurality can be associated with low birth weight and SGA (Strutz et al., 2012), also population-dense urban areas have been shown to have elevated rates of adverse birth outcomes (Kent et al., 2013). Low maternal education increases the risk for preterm birth and SGA (Ruiz et al., 2015). In our study group, women with basic education were more likely to live in rural areas (29%) than women with a university degree (10%) (Chi-Square test *P* = 0.041), which might explain the over-representation of AGA in the urban areas we have investigated. It remains to be clarified which (other) urban-specific factors in Bratislava and Vienna have contributed to birth outcome.

Three gene variants ABCC1 (rs246221; maternal genotype), GCLM (rs41303970; combined maternal-infant genotype) and HFE (rs1799945; combined maternal-infant genotype) were found to be associated with SGA. Interestingly, all three proteins are related to conditions of oxidative stress. ABCC1 has an important role in maintaining cellular redox status (Cole, 2014) also in placental cells (Granitzer et al., 2020), GCLM is the rate-limiting enzyme in the synthesis of the major cellular anti-oxidant GSH (Liu, 2013), and HFE malfunction is associated with systemic iron overload (hemochromatosis) promoting oxidative stress (De Souza et al., 2015). When combining these ABCC1, GCLM, and HFE genotypes, we found a significant over-representation of the major alleles in AGA as well as a significant over-representation of the variant alleles in SGA babies. Despite the small sample numbers we were able to analyze, the adverse effect of these gene variants on birth weight seems plausible. Oxidative stress conditions are known to be associated with lower birth weight and SGA (Weber et al., 2014). Moreover, the presence of the HFE H63D variant allele has been shown to predict a reduction of 110 g in birth weight (when infants carry the variant) and of 52 g (when mothers carry the variant) in a Pb-exposed population (Cantonwine et al., 2010).

Vitamin D deficiency during pregnancy can affect fetal growth as indicated in animal experiments (Liu et al., 2013; Wilson et al., 2015) and human studies (Harvey et al., 2014; Knabl et al., 2017). The possible mechanisms linking Vitamin D to fetal growth include calcium metabolism, bone growth, and placental functions such as human chorionic gonadotropin secretion, placental sex steroid production, and glucose transport (Knabl et al., 2017). Vitamin D activity is mediated by its receptor VDR. Among the most frequently examined VDR gene variants (FokI, rs2228570; ApaI rs7975232; Bsml rs1544410; TaqI, rs 731236), ApaI, BsmI, and TaqI genotype are in linkage disequilibrium in Caucasian populations (Knabl et al., 2017). In several studies these VDR SNPs increased the risk of preterm birth (Rosenfeld et al., 2017; Dutra et al., 2019). As determined in a meta-analysis, the here investigated VDR polymorphism (syn. BsmI, i.e., the A allele) protected from preterm delivery (Barchitta et al., 2018), which may explain why we found this VDR variant (infants carry the A allele) associated with AGA. More research on the possible protective role of this and other VDR gene variants on birth outcome is required.

### Strengths and Limitations of the Study

Our study has several strengths. We described in a first attempt the exposure situation of pregnant women and newborns for a variety of substances of high concern including 16 PFAS compounds. We also performed genotyping in both pregnant women and newborns to obtain information on the contribution of the maternal, the infant, and the combined maternal-infant genotype to placental transfer rates of PFAS, Hg, and Pb, and to SGA prevalence. In most cases, the infant genotype (alone and in combination with the maternal genotype) explained placental transfer rates and SGA prevalence better than the maternal genotype alone. This at least suggests that the infant genotype has a large influence on placental transfer rates of the here examined toxins. Further studies are necessary to clarify these relationships, also by taking into account the paternal genotype.

Considering the low to moderate exposure in the Bratislava-Vienna region, the here reported findings might not be applicable to situations of higher exposure. As small sample numbers increase the risk of false-negative findings, one further limitation of the study is that BPA, PFAS, and MeHg levels could have been measured only in a limited number of pregnant women and newborns.

## Conclusion

Fetal exposures to toxic substances and endocrine disrupters can adversely affect lifetime health. In agreement with other European studies, our results show the need to minimize perinatal exposures. Especially exposures to PFAS and Pb should be investigated at more regular intervals. With regard to Pb, because there is little recent data on exposure in Europe and the existing guidelines provide limited guidance. In case of PFAS it is necessary to evaluate the effectiveness of regulation measures as well as to avoid regrettable substitutions.

Despite of close geographical proximity, the exposure situation in Bratislava and Vienna was different. Efforts are required to identify the exposure sources to PFOA and BPA in Bratislava as well as to Pb and PFOS in Vienna. Our findings were the basis for information folders disseminated to pregnant women at clinics in Vienna and Bratislava as well as to the interested public. Follow-up studies are necessary in order to evaluate the effectiveness of such preventive measures.

We found gene sequence variants associated with placental transfer rate and with SGA prevalence. Basic research to investigate placental transfer rates of priority substances in connection with genetic factors is of utmost importance in order to define and protect genetically predisposed risk groups.

## Data Availability Statement

The datasets for this article are not publicly available due to concerns regarding participant/patient anonymity. Requests to access the datasets should be directed to the corresponding author.

## Ethics Statement

The studies involving human participants were reviewed and approved by ethic committee of the City of Vienna (EK 09-191-1109) Ethic Committee of the University clinic in Bratislava (EK-62-2010). Written informed consent to participate in this study was obtained from the study participants or the participants’ legal guardians/relatives.

## Author Contributions

MU, MG, and CG: funding acquisition and writing–original draft. MU, CG, KG-R, and MG: study concept and design. KH and PR: recruitment of study participants and sampling (University Hospital Bratislava). KG-R, TR, and IT: recruitment of study participants and sampling (Semmelweis Klinik Vienna). ZD, MG, RK, VP, TR, IT, and WR: data collection. TR, IT, CG, SS, BK-V, and SW: analytics. CG, IO, MH, ZD, and MG: statistics and data interpretation. MG, MH, KG-R, KH, and SS: resources. CG, MH, MU, ZD, RK, IO, VP, and KG-R: writing–review and editing. All authors approved the final version of the manuscript.

## Conflict of Interest

The authors declare that the research was conducted in the absence of any commercial or financial relationships that could be construed as a potential conflict of interest.

## Footnotes

1 UmMuKi stands for Umwelt-Mutter-Kind (environment-mother-child).

2 REACH stands for the Registration, Evaluation, Authorisation and Restriction of Chemicals.

3 http://www.oecd.org/chemicalsafety/portal-perfluorinated-chemicals/ [accessed 31 Jan 2021].

4 https://echa.europa.eu/hot-topics/bisphenol-a. [accessed 31 Jan 2021].

5 http://www.cdc.gov/nceh/lead/ACCLPP/Final_Document_030712.pdf [accessed 31 Jan 2021].

## Supplementary Material

Supplementary Table 1 Correlation matrix of PFAS levels (sum parameter^§^ and single compounds) in 42 cord serum and maternal serum samples each.

Supplementary Table 2 Correlation matrix of THg, MeHg and Pb levels.

Supplementary Table 3 Factors associated with PFAS^§^ levels in CATREG analysis.

Supplementary Table 4 Associations of exposure factors with THg levels in CATREG analysis.

Supplementary Table 5 Factors associated with Pb levels in CATREG analysis.
