# Supplementary material for: Gene Variants Determine Placental Transfer of Perfluoroalkyl Substances (PFAS), Mercury (Hg) and Lead (Pb), and Birth Outcome: Findings From the UmMuKi Bratislava-Vienna Study
Source: Front Genet. 2021 Jun 16;12:664946. doi: 10.3389/fgene.2021.664946 (PMC8242356; doi:10.3389/fgene.2021.664946)
Supplement: Supplementary Table 1 — Correlation matrix of PFAS levels (sum parameter§ and single compounds) in 42 cord serum and maternal serum samples each. [file Data_Sheet_1.doc]

**Supplemental data**

***Gene variants determine placental transfer of perfluoroalkyl substances (PFAS), mercury (Hg) and lead (Pb), and birth outcome: Findings from the UmMuKi Bratislava-Vienna study***

Claudia Gundacker1†*, Klaudia Graf-Rohrmeister2†, Martin Gencik3, Lucia Behalova3, Markus Hengstschläger1, Karol Holoman4, Petra Rosa4, Renate Kroismayr5,6, Ivo Offenthaler5, Veronika Plichta1, Theresa Reischer1, Isabella Teufl1, Wolfgang Raffesberg5, Sigrid Scharf5, Birgit Köhler Vallant5, Zoja Delissen1,3, Stefan Weiß5, Maria Uhl5

**Suppl. Table 1.** Correlation matrix of PFAS levels (sum parameter^§^ and single compounds) in 42 cord serum and maternal serum samples each

*P<0.05, **P<0.01,***P<0.001, Spearman correlation

CordS: umbilical cord serum; MatS: maternal serum

^§^PFAS sum parameter of PFOS, PFOA, PFNA, PFHxS, PFDA, PFUnDA, PFBA, PFPeA, PFHxA PFHpA, PFDoA, PFTrDA, PFTeDA, PFBS, PFHptS, PFDS concentrations

**Suppl. Table 2**. Correlation matrix of THg, MeHg and Pb levels

|  |  | **CordEry-THg (µg/kg)** | **MatEry-THg (µg/kg)** | **ChildBl-MeHg (µg/l)** | **MatBl-MeHg (µg/l)** | **CordEry-Pb (µg/kg)** |
| --- | --- | --- | --- | --- | --- | --- |
|  |  |  |  |  |  |  |
| MatEry-THg (µg/kg) | r | **0.717^**^** |  |  |  |  |
|  | P | <0.001 |  |  |  |  |
|  | N | 172 |  |  |  |  |
| ChildBl-MeHg (µg/l) | r | **0.811^**^** | **0.703^**^** |  |  |  |
|  | P | <0.001 | <0.001 |  |  |  |
|  | N | 40 | 33 |  |  |  |
| MatBl-MeHg (µg/l) | r | **0.778^**^** | **0.604^**^** | **0.900^**^** |  |  |
|  | P | <0.001 | <0.001 | <0.001 |  |  |
|  | N | 40 | 33 | 40 |  |  |
| CordEry-Pb (µg/kg) | r | **0.239^**^** | 0.026 | 0.117 | 0.273 |  |
|  | P | 0.001 | 0.723 | 0.472 | 0.088 |  |
|  | N | 189 | 182 | 40 | 40 |  |
| MatEry-Pb (µg/kg) | r | 0.138 | -0.008 | **0.424^**^** | **0.456^**^** | **0.646^**^** |
|  | P | 0.060 | 0.915 | 0.007 | 0.004 | <0.001 |
|  | N | 187 | 182 | 39 | 39 | 198 |
|  |  |  |  |  |  |  |

**P<0.01, Spearman correlation

CordEry: umbilical cord erythrocytes; MatEry: maternal erythrocytes, CordBl: umbilical cord whole blood, MatBl: maternal whole blood

**Suppl. Table 3.** Factors associated with PFAS^§^ levels in CATREG analysis

**Exposure Factor Partial r Importance**

**Marker [model] β ± S.E.^a^ P [R^2^] coeff. (rank)**

MatS-PFAS Study site 0.470 ± 0.107 <0.001 0.501 0.677 (1)

(µg/l) Parity -0.312 ± 0.104 0.005 -0.359 0.323 (2)

[Crude model with 3 factors]^c^ <0.001 [0.392]

[Final model with 2 factors] <0.001 [0.348]

CordS-PFAS MatS-PFAS 0.809 ± 0.060 <0.001 0.850 0.902 (1)

(µg/l) Gestational length 0.219 ± 0.093 0.024 0.400 0.098 (2)

[Crude model with 5 factors]^b^ <0.001 [0.757]

[Final model with 2 factors] <0.001 [0.753]

^a^Standardized slope ± standard error

^b^The crude model included 5 factors (significant in bivariate statistics), i.e. MatS-PFAS (µg/L), gestational length (d), parity (no. of pregnancies), maternal chronic disease (1=no, 2=yes), cord blood hemoglobin (g/dl)

^c^The crude model included 3 factors (significant in bivariate statistics), i.e. parity (no. of pregnancies), study site (1=Vienna, 2=Bratislava), maternal chronic disease (1=no, 2=yes)

^§^PFAS: sum parameter of PFOS, PFOA, PFNA, PFHxS, PFDA, PFUnDA, PFBA, PFPeA, PFHxA PFHpA, PFDoA, PFTrDA, PFTeDA, PFBS, PFHptS, PFDS concentrations

**Suppl. Table 4.** Associations of exposure factors with THg levels in CATREG analysis

**Exposure Factor Partial r Importance**

**Marker [model] β ± S.E.^a^ P [R^2^] coeff. (rank)**

**MatEry-THg** Fish consumption 0.472 ± 0.056 <0.001 0.672 0.865 (1)

(µg/kg) No. of amalgam fillings 0.357 ± 0.059 <0.001 0.225 0.135 (2)

[Crude model with 4 factors]^c^ <0.001 [0.309]

[Final model with 2 factors] <0.001 [0.304]

**CordEry-THg** MatEry-THg 0.663 ± 0.051 <0.001 0.433 0.877 (1)

(µg/kg) Fish consumption 0.169 ± 0.055 0.002 0.152 0.123 (2)

[Crude model with 6 factors]^b^ <0.001 [0.592]

[Final model with 2 factors] <0.001 [0.558]

^a^Standardized slope ± standard error

^b^ The crude model included 6 factors (significant in bivariate statistics), i.e. MatEry-Hg (µg/kg), study site (1=Vienna, 2=Bratislava), maternal fish consumption (g/week), maternal education level (highest completed education coded 1: grammar school, 2: higher education college, master profession, 3: university), maternal age (yrs), neonate sex (1: female, 2: male)

^d^The crude model included 4 factors (significant in bivariate statistics), i.e. maternal fish consumption (g/week), no. of amalgam fillings, maternal education level (highest completed education coded 1: grammar school, 2: higher education college, master profession, 3: university), maternal age (yrs)

**Suppl. Table 5.** Factors associated with Pb levels in CATREG analysis

**Exposure Factor Partial r Importance**

**marker [model] β ± SE^a^ P [R^2^] coeff. (rank)**

**MatEry-Pb** Study site 0.229 ± 0.066 0.001 0.235 0.312 (1)

(µg/kg) Age of residential building 0.243 ± 0.078 0.002 0.252 0.291 (2)

Maternal age 0.239 ± 0.065 <0.001 0.269 0.205 (3)

Years of smoking 0.199 ± 0.083 0.004 0.224 0.192 (4)

[Crude model=Final model] <0.001 [0.277]

**CordEry-Pb** MatEry-Pb 0.596 ± 0.045 <0.001 0.628 0.770 (1)

(µg/kg) Study site 0.213 ± 0.051 <0.001 0.283 0.165 (2)

Maternal age 0.131 ± 0.050 0.009 0.185 0.065 (3)

[Crude model with 7 factors]^b^ <0.001 [0.572]

[Final model with 3 factors] <0.001 [0.536]

^a^Standardized slope ± standard error

^c^The crude model included 7 factors (significant in bivariate statistics), i.e. MatEry-Pb (µg/kg), study site (1=Vienna, 2=Bratislava), maternal age (yrs), age of residential building (1: constructed before 1945, 2: constructed after 1945), maternal years of smoking, maternal hemoglobin (g/dl), cord blood hemoglobin (g/dl)
